# Expression and Localization of BDNF/TrkB System in the Zebrafish Inner Ear

**DOI:** 10.3390/ijms21165787

**Published:** 2020-08-12

**Authors:** Antonino Germanà, Maria Cristina Guerrera, Rosaria Laurà, Maria Levanti, Marialuisa Aragona, Kamel Mhalhel, Germana Germanà, Giuseppe Montalbano, Francesco Abbate

**Affiliations:** Zebrafish Neuromorphology Lab, Department of Veterinary Sciences, University of Messina, 98168 Messina, Italy; agermana@unime.it (A.G.); laurar@unime.it (R.L.); mblevanti@unime.it (M.L.); mlaragona@unime.it (M.A.); kmhalhel@unime.it (K.M.); pgermana@unime.it (G.G.); gmontalbano@unime.it (G.M.); abbatef@unime.it (F.A.)

**Keywords:** brain-derived neurotrophic factor, TrkB, inner ear, development, zebrafish

## Abstract

Brain-derived neurotrophic factor (BDNF), a member of the neurotrophin family, is involved in multiple and fundamental functions of the central and peripheral nervous systems including sensory organs. Despite recent advances in knowledge on the functional significance of BDNF and TrkB in the regulation of the acoustic system of mammals, the localization of BDNF/TrkB system in the inner ear of zebrafish during development, is not well known. Therefore, the goal of the present study is to analyze the age-dependent changes using RT-PCR, Western Blot and single and double immunofluorescence of the BDNF and its specific receptor in the zebrafish inner ear. The results showed the mRNA expression and the cell localization of BDNF and TrkB in the hair cells of the crista ampullaris and in the neuroepithelium of the utricle, saccule and macula lagena, analyzed at different ages. Our results demonstrate that the BDNF/TrkB system is present in the sensory cells of the inner ear, during whole life. Therefore, this system might play a key role in the development and maintenance of the hair cells in adults, suggesting that the zebrafish inner ear represents an interesting model to study the involvement of the neurotrophins in the biology of sensory cells

## 1. Introduction

According to the World Health Organization, around 466 million people (5% of the populations) worldwide have disabling hearing loss. The onset of the hearing disorders originate from genetic problems to pharmacological treatments, through infectious diseases and exposure to strong noise. Particularly, the non-sensorineural hearing loss represents the most common hearing disorder, caused by a serious morphofunctional alterations of cochlear hair cells. Brain-derived neurotrophic factor (BDNF) belongs to the neurotrophin family and is involved in the development, maintenance and neuronal plasticity of the different neuronal subpopulations of the central and peripheral nervous systems [1]. BDNF acts on cell surfaces through a specific receptor called TrkB. Brain-derived neurotrophic factor (BDNF) and neurotrophin 3 (NT-3) with their specific receptors (TrkB and TrkC) play a key role in the regulation of the acoustic system of mammals [2]. Recently, the expression and localization of neurotrophins and their receptors have been found in the inner ear and lateral line system of different teleosts [3,4,5]. It was demonstrated that the BDNF and TrkB sequences are well preserved during evolution [6] and therefore these two proteins have been identified and analyzed thoroughly in the fish nervous system with a specific focus on the zebrafish sensory organs. BDNF and its specific receptor TrkB were localized in different areas of both adult and developing zebrafish brain [7,8,9,10,11], in the photoreceptor layer of the retina, from larval to adult stage, under both physiological and experimental conditions with an identical pattern of distribution and cell localization [12], in the hair cells of the lateral line system during development [13] and in the chemosensory cells of the taste buds [14]. In teleosts, the auditory system is formed by the inner ear and lateral line system. The inner ear, more specifically, consists of the labyrinth formed by three semicircular canals, connected to a sacciform structure, the utricle, which connects, in the lower part, to a second sacciform structure, the saccule. Adjacent to the saccule is the lagena. At the base of each canal, the bony region is enlarged and has a dilated sac at one end, called the osseous ampullae. Each ampulla contains a patch of sensory epithelium with an evident round shape, called crista ampullaris. Utricle, saccule and lagena are provided with a thickening of sensory cells called macula, whose kinocilia and stereocilia are connected to dense limestone structures, the otoliths [15]. It is well-known that the utricle is usually considered to be a vestibular organ; the sacculus is involved in sound reception and the lagena assists with sacculus functions playing an important role in orientation and hearing too [16]. The zebrafish is now recognized as an important experimental animal for studying developmental biology and genetics as well as for modeling human disorders including sensorineural hearing loss mainly resulting from damage to the sensory hair cells of the inner ear [17,18,19]. The hair cells of the zebrafish inner ear present structural and molecular homology with the sensory cells of the mammalian inner ear including that of humans. Moreover, numerous genes as ATOH1, POU4F3, GFI1 and other genes, are expressed in the sensory cells of zebrafish, mainly during embryonic development, also expressed in mammals [20]. It is well known that zebrafish is able to regenerate, within a few days, the hair cells damaged or lost by acoustic trauma, chemical exposure or genetics [21,22,23,24,25]. Therefore, based on the peculiar ability of zebrafish to regenerate new hair cells, this study was undertaken to analyze the cellular localization of BDNF and its specific receptor TrkB location in the inner ear of the zebrafish. 

## 2. Results

### 2.1. Anatomical Study of Zebrafish Inner Ear

**Figure 1:**
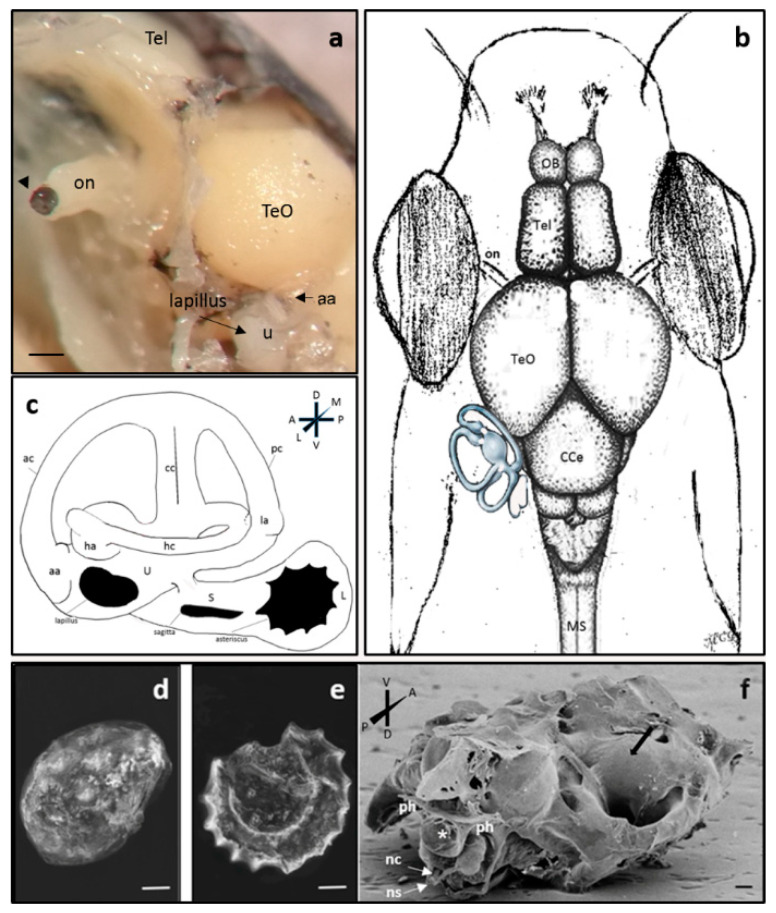
(**a**) Stereomicrograph: Cranial cavity dissection in adult zebrafish, lateral view: the bone skull has been removed in order to highlight the brain and the topographical relationships with the inner ear; some bony labyrinth was dissected away to improve visualization of membranous labyrinth: Tel—Telencephalon; TeO—Optic tectum; on—optic nerve with optic papilla (arrowhead) u—utricle, lapillus in the utricle, aa—anterior ampulla; (**b**) schematic drawing of the dorsal view zebrafish head without the skull: the topographical relationship of the inner ear with respect to TeO and eyes is represented. OB—olfactory bulb, Tel—Telencephalon, TeO—Optic tectum, CCe—cerebellar corpus, MS—spinal cord. (**c**) schematic drawing of the membranous labyrinth in adult zebrafish: lateral view; Orientation: D-dorsal, V-ventral, A—anterior, P—posterior, L—lateral, M—medial; aa—anterior ampulla; ac—anterior canal; cc—crus commune; ha—horizontal ampulla; hc—horizontal canal; la—lateral ampulla; pc—posterior canal; S—saccule; U—utricle; L—lagena. The otoliths (lapillus, sagitta, asteriscus) have been depicted in the respective otolithic organs, utricle, saccule and lagena. (**d**–**f**) Scanning electron microscopy: lapillus (**d**), asteriscus (**e**), base of the neurocranium, ventro-dorsal view: cervical vertebra in posterior view (asterisk) (**f**). The black arrow shows a landmark of the inner ear area placed inside the bone capsule; ph—parapophysis, nc—neural canal, ns—neural spine. Scale bar: (**a**) 1 mm, (**d**–**f**) 100 μm.

The inner ear in zebrafish is located in a bone capsule in the cranial cavity, posterolaterally to the optic tectum, just behind the eyes (Figure 1a,b). It is divided into an upper part including three semicircular canals related to the utricle and the lower part made up of the saccule and lagena, also called otolith organs. The semicircular canals are spatially oriented in three mutually perpendicular planes following the main axis and orthogonally among them. The anterior and posterior canals are placed vertically and the lateral canal horizontally. The opposite parts of the anterior and posterior canals considering the ampullary end, converge in a common part called crus communis (Figure 1).

**Figure 2:**
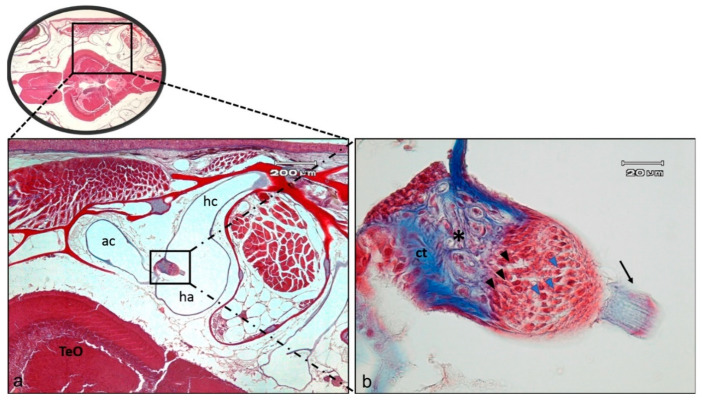
(**a**) Light micrographs (Masson Trichrome with Anylin blue staining) of adult zebrafish head; dorso-ventral view, horizontal section: semicircular horizontal canal (hc) of the inner ear, with its ampulla (ha) containing the crista ampullaris, placed near Optic tectum (TeO), are visible; ac—semicircular anterior canal; (**b**) High magnification of ampullary crest in the horizontal canal: the connective tissue (ct) supports nerve fibers (asterisk). The black arrowheads indicate the supporting cells, blue arrowheads indicate the hair cells, the arrow points to the cupula.

At the end of each semicircular canals a dilated sac is present, called ampulla ossea, containing a cluster of sensory cells called crista ampullaris and a thick gelatinous cap called cupula (Figure 2a). The crista ampullaris shows a cuboidal epithelium. In the apical part a sensory epithelium with hair, supporting and basal cells is present (Figure 2b).

**Figure 3:**
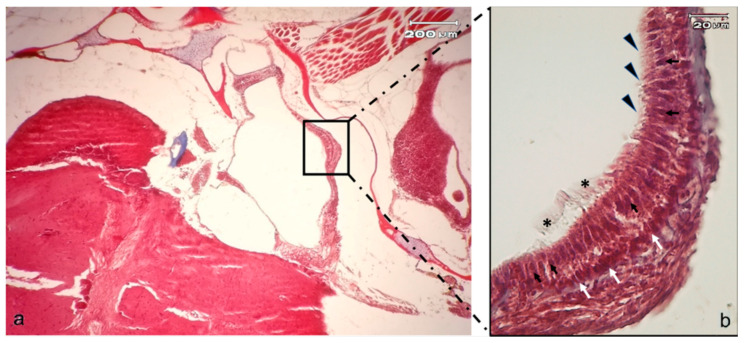
(**a**) Light micrographs (Masson Trichrome with Anylin blue staining) of adult zebrafish head; dorso-ventral view, horizontal section: macula of utricle (rectangle); (**b**) High magnification of utricular macula. The portion of the utricle that forms the macula shows a sort of pouch. The sensory hair cells (black arrows) with numerous stereocilia (arrowheads) and less numerous but longer kinocilia (asterisk) are visible. The white arrows indicate the supporting cells.

Each otolith organ, containing an oval thickening called maculae of supporting cells, interdigitates among hair sensory cells (Figure 3). 

**Figure 4:**
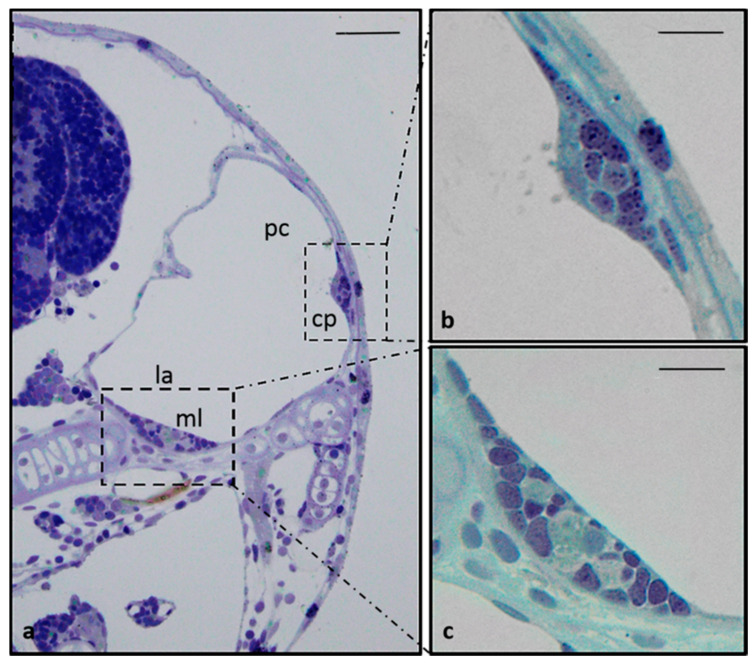
(**a**) Transverse section of the larval stage head (8 dpf), semithin section, toluidine blue staining. Structure of an 8 days old inner ear: crista ampullaris (square) and macula (rectangle). (**b**) High magnification of sensory epithelial cells of lateral cristae: (**c**) High magnification saccular macula. ml—macula lagena; la—lagena; pc—posterior canal, cp—crista posterior. Scale bars: (**a**) 30µm, (**b**,**c**) 9 µm.

Dense calcareous structures are present in close proximity to sensory epithelium, the utricular otolith (lapillus), saccular otolith (arrow) and lagenar otolith (asterisk) located in a small cavity in the temporal bone (Figure 1d–f). In the larval stage, at 8 days post fertilization (dpf), the pars inferior shows little resemblance to the final adult form but is merely a slight ventromedial pouch containing the posterior macula (Figure 4).

Although its dimensions have increased, the 15, 30 and 50 dpf ear is characterized by the semicircular canals and utricle, and the anatomical spatial organization and the histological features of the neuroepithelium are very similar to the adult zebrafish (data not shown). 

### 2.2. Expression and Occurrence of Bdnf and TrkB in Zebrafish Inner Ear

**Figure 5:**
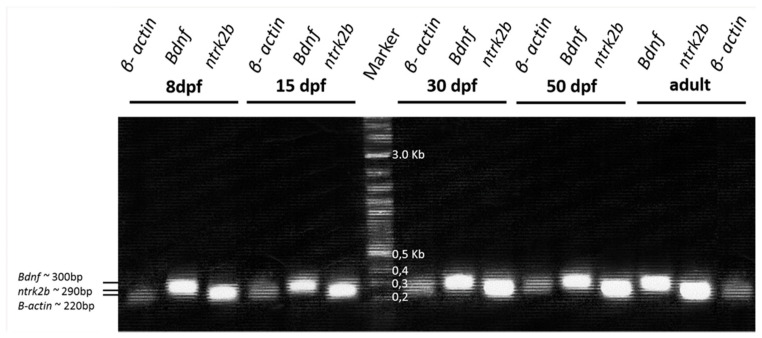
RT-PCR, *Bdnf* and *ntrk2b* mRNA expression in the inner ear from larval to adult stage. *B-actin* controls for each condition are also shown. Lines 1, 2 and 3: detection of *β-actin, Bdnf* and *ntrk2b* at 8 dpf. Lines 4, 5 and 6: detection of *β-actin*, *Bdnf* and *ntrk2b* at 15 dpf. Line 7 Marker. Lines 8, 9 and 10: detection of *β-actin*, *Bdnf* and *ntrk2b* at 30 dpf. Lines 11, 12 and 13: detection of *β-actin, Bdnf* and *ntrk2b* at 50 dpf. Lines 14, 15 and 16: *Bdnf, ntrk2b and β-actin* in the adult stage. The size of the amplified fragment is indicated.

The expression of *Bdnf* and *ntrk2b* mRNA was assayed in the homogenates of zebrafish inner ear using reverse transcriptase PCR. Our observations demonstrated that *Bdnf* and *ntrk2b* are expressed at 8, 15, 30, 50 dpf and adult stage. *B-actin* controls for each condition were also conducted (Figure 5). 

**Figure 6:**
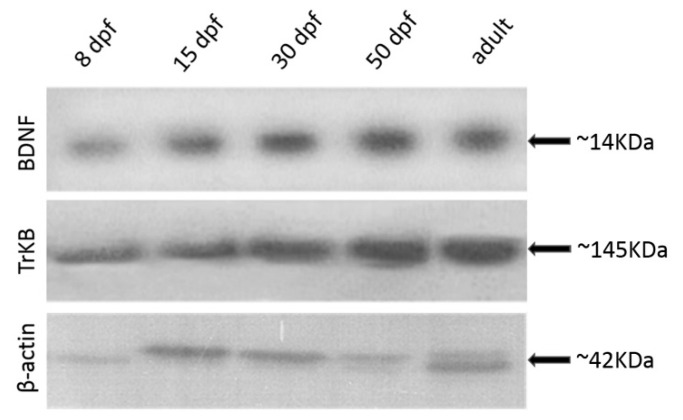
Western blot: detection of BDNF at 8 dpf (line 1), 15 dpf (line 2), 30 dpf (line 3), 50 dpf (line 4), adult stage (line 5). Detection of TrKB at 8 dpf (line 1), 15 dpf (line 2), 30 dpf (line 3), 50 dpf (line 4), adult stage (line 5). β-Actin protein was used as an endogenous control: 8 dpf (line 1), 15 dpf (line 2), 30 dpf (line 3), 50 dpf (line 4), adult stage (line 5).

The negative (not shown) and positive controls were performed to validate the obtained results. Moreover, in order to test the specificity of the antibodies utilized, we performed western blot analysis at the same age. Two specific protein bands with a molecular weight of 14 and 145 kDa, corresponding to the mammalian isoform of BDNF and TrkB respectively, were observed. β-Actin protein was used as an endogenous control to allow the normalization of BDNF and TrKB proteins (Figure 6).

### 2.3. Immunofluorescence

**Figure 7:**
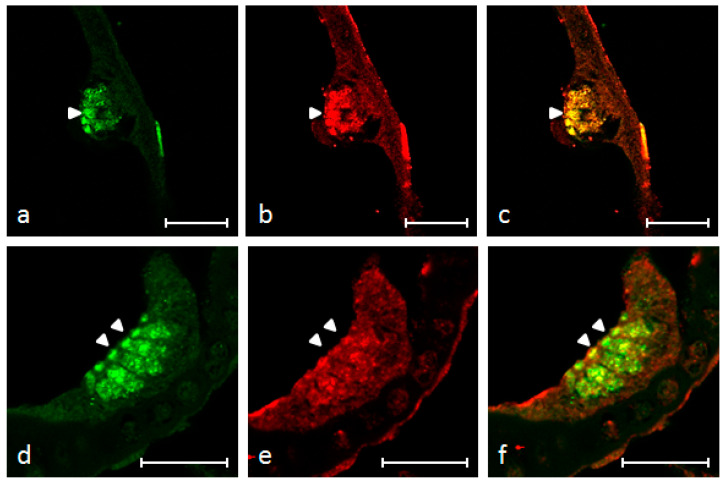
Inner ear of zebrafish larva 8 dpf: immunohistochemical detection of proBDNF (**a**), trkB (**b**) and colocalization of both antibodies (**c**) in utricular macula; Immunohistochemical detection of proBDNF (**d**), trkB (**e**) and colocalization of both antibodies (**f**) in lateral crista. Immunoreactivity was found in the sensory epithelial cells of utricular macula and lateral crista. Scale bars = 20 μm.

An immunohistochemical analysis was carried out in serial sections using single and double immunofluorescence. Cellular localization was performed in zebrafish larvae using double immunofluorescence with a monoclonal antibody against pro-BDNF and a polyclonal antibody against TrkB. Moreover, in order to identify the positive cells, we used a morpho-topographical approach based on the observation of the cellular histological features. The results, using confocal laser microscopy, demonstrated that pro-BDNF and TrkB were found only in the hair cells of the macule and cristae lateralis with an identical pattern of expression (Figure 7a–f). To make clear what is signal and what is background, both negative and positive controls are provided (Supplementary Materials).

**Figure 8:**
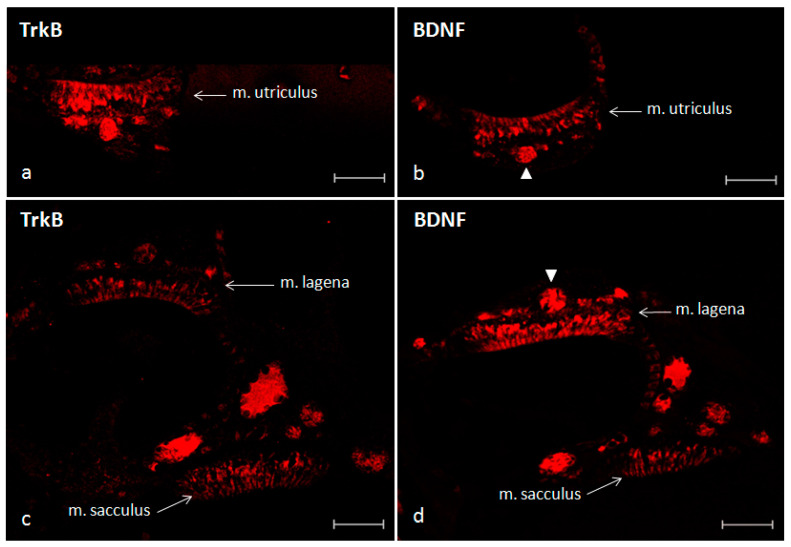
Inner ear of adult zebrafish. Immunohistochemical localization of TrkB (**a**) and BDNF (**b**) in the macula of the utricle. Immunohistochemical localization of TrkB (**c**) and BDNF (**d**) in the macula of the lagena and saccule. Arrowheads in (**b**,**d**) indicate nerves. Scale bars = 50 μm.

Regarding the adult zebrafish, an intense and strong immunostaining for BDNF, and its specific receptor TrkB, was observed in the maculae of the utricle, saccule and lagena and more specifically in the hair bundle of the sensory cells (Figure 8a,b).

**Figure 9:**
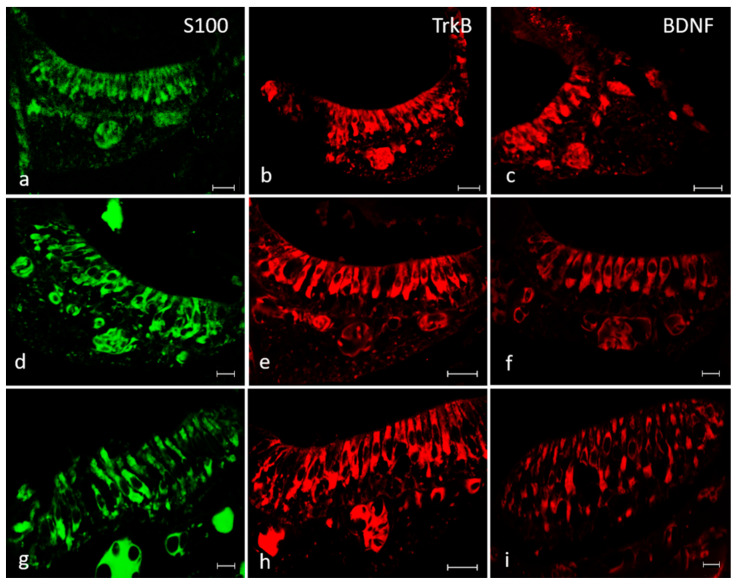
Inner ear of adult zebrafish. Immunohistochemical detection of S100 protein* in the macula of the utricle (**a**), saccule (**d**) and lagena (**g**), of TrkB in the macula of the utricle (**b**), saccule (**e**) and lagena (**h**). Immunohistochemical detection of BDNF in the macula of the utricle (**c**), saccule (**f**) and lagena (**i**). The utricular and saccular maculae consist of a cylindrical epithelium formed by sensory hair, supporting, mantle and basal cells. Cytoplasmic immunoreactivity for these antibodies was found in the sensory epithelial cells of the utriculus, sacculus and lagena. Scale bars = 10 μm (**a**,**c**,**d**,**f**,**g**,**i**). Scale bar s = 20 μm (**b**,**e**,**h**). The authors specify that the green channel was chosen to differentiate the immunofluorescence for protein S100.

Moreover, the immunohistochemical detection performed in serial sections, also using S100 protein as specific marker for hair cells in order to identify the cells displaying BDNF and TrkB, demonstrated a similar pattern of distribution (Figure 9). Both BDNF and TrkB were localized in the cytoplasm of cylindrical cells placed in the apical part of the sensory patches of the different macules. These cells were identified as sensory hair cells because of their morphology and localization as well as their S100 protein immunoreactivity (Figure 9). In fact, S100 proteins are a large subfamily of EF-hand Ca2+-binding proteins localized in the cytoplasm and/or nucleus of a wide range of cells, participating in the regulation of intracellular Ca2+ homeostasis as trigger or activator proteins. S-100 immunoreactivity, in previous studies, has been localized within the sensory epithelium of the saccular macula, hair cells and myelinated saccular nerve fibers [14,25,26,27,28,29,30].

**Figure 10:**
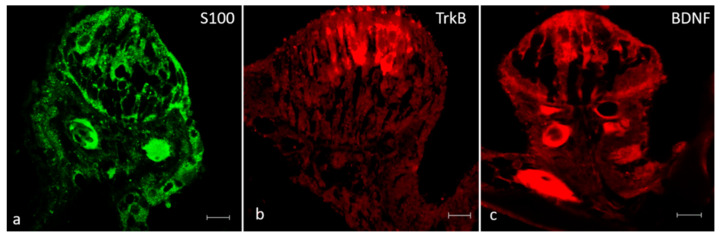
Inner ear of adult zebrafish. The cristae ampullaris in the apical portion contain sensory hair, supporting and basal cells. The sensory hair cells of the cristae ampullaris displayed cytoplasmic immunoreactivity for S100 protein. (**a**), TrkB (**b**) and BDNF (**c**). Scale bars= 10 μm. The authors specify that the green channel was chosen to differentiate the immunofluorescence for protein S100.

In addition, BDNF, TrkB and S100 proteins were detected in the cristae ampullaris of the semicircular canals. Specific staining was found in a subpopulation of hair cells localized in the central and peripheral part of the sensory cells cluster respectively (Figure 10).

## 3. Discussion

This study demonstrates the expression and cell localization of the BDNF, and its specific receptor TrkB in the inner ear of zebrafish from larval to adult stages. Moreover, the S100 protein was also found in the sensory cells of the zebrafish inner ear. This finding is in complete agreement with previous results, demonstrating the presence and specificity of the S100 protein used as a specific marker for the identification of hair cells in the inner ear and lateral line system of zebrafish at different stages of development [14,26,27,28,29,30]. The expression and the presence of the BDNF and TrkB at mRNA and protein levels and a specific immunoreactivity for the BNDF and TrkB in the sensory cells of the inner ear were found [31]. The specificity of the antibodies used in this study has been also previously demonstrated by our research group using cross pre-absorption analysis and the western blot technique on homogenates of the whole head of the zebrafish [12,13,32]. It is well known that neurotrophins are involved in the development and maintenance of the auditory system. Particularly, BDNF is expressed in sensory epithelium of the utricle and saccule of mammals, birds and amphibians [33,34,35]. The data, present in literature, regarding the presence and distribution of BDNF and TrkB in the inner ear of zebrafish, is scarce and there are only a few reports related to the expression of neurotrophins and their specific receptors in the sensory organs of different teleosts [3,4,5]. Specifically, BDNF and TrkB in zebrafish are expressed and localized in the mechanosensory cells of the lateral line system from embryo to adult stage [13]. BDNF and TrkB in the sensory patches of both macula and crista ampullaris were found. These findings are in part consistent with previous studies performed in mammals where the BDNF is restricted only to the hair cells of maculae and to the crista ampullaris [2]. Neurotrophins and their receptors including BDNF and TrkB have been well preserved during vertebrate evolution [36]. TrkB also known as neurotrophic tyrosine kinase receptor (ntrk) has two isoforms in zebrafish, ntrk2a and ntrk2b. In this study we investigate the expression of ntrk2b because it is the most expressed isoform in mechanosensory systems, if compared to ntrk2a [13,37]. In the last decades, several animal models, including zebrafish, have been used in biomedicine in order to model sensorineural hearing loss and study the morpho-functional alteration and ototoxic lesions due to pharmacological treatments mainly carried out with aminoglycoside antibiotics in the sensory cells of the inner ear [38,39]. It is well known that the spontaneous regeneration of hair cells is not possible in mammals whereas fish maintain the capacity to regenerate damaged hair cells within a few days [25,40,41]. Moreover, we demonstrated that the anatomical structures and the histological aspects of the inner ear neuroepithelium in fish resemble those observed in mammals [42]. In vertebrates, the neurotrophins play an important role during the development and maintenance of the auditory system in adult stage. Recent findings have showed that sensorineural hearing loss leads to an evident reduction of the cochlear hair cells with a decrease of the spiral ganglion neurons probably due to less availability of neurotrophic factors. However, the exogenous treatment with BDNF and NT-3 partially restores the loss of cochlear hair cells [43,44] and also implements neurite outgrowth [45]. The findings that the BDNF/TrkB system is present in the sensory patches of the inner ear during the whole life cycle, the morpho-functional and molecular analogy, the similarity of the localization pathway as well as the role of the BNDF/TrkB system in the biology of the auditory system, both in zebrafish and mammals, indicates a possible important role of this complex in the development, maintenance and mainly in the regenerative process of the hair cells in zebrafish. Finally, based on the obtained results, we can assume that the zebrafish inner ear represents a perfect model to study the growth factors in the biology of sensory cells with special attention to the regenerative events within the sensory patches of the inner ear. Studies are in progress in our laboratory targeted at creating mutant zebrafish for BDNF and TrkB to deeply analyze the functional activity of BDNF in the mechanical-sensory organs of zebrafish

## 4. Materials and Methods 

### 4.1. Zebrafish Breeding and Tissue Treatments

In this study, we used eighty (80) zebrafish from larval to adult stage. Particularly, we utilized samples at 8 days post-fertilization (dpf), 15 dpf, 30 dpf, 50 dpf and adulthood. The fish were obtained from CISS (Center of Experimental Ichthyiopathology of Sicily, University of Messina, Italy) and kept on a 14 h day, 10 h night cycle at a constant temperature of 28.5 °C and were fed twice a day. All embryos were collected after natural spawning and staged according to Kimmel et al. [46]. The fishes, at the above-mentioned stage, were sacrificed with a lethal dose of tricaine methane sulfonate (MS222; 1000–10,000 mg L-1. The heads of 5 zebrafish for each group were quickly removed, fixed in 4% paraformaldehyde in phosphate buffered saline (PBS) 0.1 m (pH = 7.4) for 12–18 h, dehydrated trough graded ethanol series, clarified in xylene, for paraffin wax embedding. Included tissues were then cut in to 7 μm thick serial sections and collected on gelatin-coated microscope slides and then routinely processed for histological and immunohistochemical analysis. Furthermore, three adult and two larval zebrafish heads (8 dpf) were fixed in 2.5% glutaraldehyde in 0.1 M Phosphate buffer, and processed, partly for scanning electron and stereo microscopy analysis and partly embedded in epoxy resin for histological study using semi-thin section (0.99 μm) stained with toluidine blue [47]. In the remaining animals (50) the inner ear was dissected and used to isolate mRNA (25) and proteins (25); there were 5 zebrafish heads for each group. The heads have been deprived of the brain, the gills, the snout including the olfactory system and the eyes and then collected in a pool per age group.

### 4.2. RT-PCR

For the isolation of RNA and Reverse Transcription PCR, total RNAs were extracted using Trizol reagent (Sigma; St. Louis, MO, USA). The integrity of RNA was checked using agarose gel electrophoresis. RNA extracted was reverse-transcribed in a final volume of 20 µL using 20 U of Superscript RNA-ase H2 Reverse Transcriptase (Gibco BRL, Gaithersburg, MD, USA) in the manufacturer’s buffer containing 2 µg RNA, 5 µM oligo (dT), 12–18 mM dNTPs 40 U RNA-ase inhibitor (Amersham Pharmacia Biotech, Little Chalfont, Buckinghamshire, UK), 0.1 µg/µL BSA and 10 mM DTT. The reaction took place at 42 °C for 90 min. The sequences of the oligonucleotide primers were based upon the published sequences for, *Brachidanio rerio* bdnf (GenBank accession number NM_131595) *Brachidanio rerio* ntrk2b (GenBank accession number NM_001197161.2) and *Brachidanio rerio* β-actin (GenBank accession number NM_131031) and were: bdnf forward: 5′AACTCCAAAGGATCCGCTCA3′, reverse: 5′GCAGCTCTCATGCAACTGA3′, for ntrk2b forward: 5′ACGAGG-ACCACATGAAGTTC3′, reverse: 5′GCAGAACGTCTCTTTCACTG3′ and for β-actin forward: 5′ CACAGATCATGTTCGAGACC3′, reverse 5′GGTCAGGATCTTCATCAGGT3′. The conditions of amplification were as follows: 2 U Taq DNA Polymerase (Promega, Madison, WI), 1 µM primers, 10 ng zebrafish brain cDNA, 0.2 mM each dNTP in 15 µLTaq DNA Polymerase buffer. The reaction was performed in a thermal cycler (Hyband Th. Cycler) with the following program: 1 min at 94 °C initial denaturation, then 10 cycles of 94 °C for 1 min, 65 °C for 30 s and 72 °C for 45 s, followed by 20 cycles of 94 °C for 1 min, 61 °C for 30 s, 72 °C for 45 s and a 5 min final extension at 72 °C. The PCR products were visualized by ethidium bromide staining under UV light following electrophoresis on a 2% agarose gel.

### 4.3. Western Blot

Frozen material was processed for Western blot. Experiments were performed in triplicate as follows: they were rinsed in cold saline, then pooled and homogenized (1:2, *w*/*v*) with a Potter homogenizer in Tris–HCl buffered saline (Tris-HCl 0.1 M, pH = 7.5) containing 1 µM leupeptin, 10 µM pepstatin and 2 mM phenylmethylsulfonyl fluoride. The homogenates were then centrifuged at 25,000× *g* for 15 min at 4 °C, and the resulting pellet dissolved in 10 mM Tris–HCl, pH = 6.8, 2% SDS, 100 mM DTT, and 10% glycerol at 4 °C. The pellets were thawed and analyzed by electrophoresis in 10% (for Trks) or 15% (for NTs) polyacrylamide SDS gels. After electrophoresis, proteins were transferred to a nitrocellulose membrane and unspecific binding was blocked by incubation for 3 h in phosphate-buffered saline containing 5% dry milk, and 0.1% Tween 20. The membranes were then incubated at 4 °C for 2 h with primary antibodies against BDNF and TrkB proteins. We used rabbit polyclonal antibodies against an amino-terminal sequence of mouse BDNF (sequence H2N-HSDPARRGEL-COOH; dilution 1:500; Chemicon International Inc., Temecula, CA, USA; catalog #AB1534SP) and TrkB (dilution 1:500, directed against the residues 794–808 of the intracytoplasmatic domain of human TrkB; Santa Cruz Biotechnology, Santa Cruz, CA, USA; catalog #sc-12). These antibodies have been characterized elsewhere for use in zebrafish and are suitable for use in Western blot and immunohistochemistry [5,13]. β-Actin protein was used as an endogenous control to allow the normalization of BDNF and TrKB proteins. We used mouse monoclonal beta Actin antibody (GT5512), validated in WB and also tested in Zebrafish (dilution 1:500, Gene Tex, Cat No. GTX629630) After incubation the membranes were washed with TBS pH = 7.6 containing 20% Tween 20, and incubated at room temperature for 1 h with goat anti-rabbit IgG secondary antibodies diluted 1:100. Membranes were washed again and incubated with the plasmin-alpha-2-antiplasmin (PAP) complex diluted 1: 100 for 1 h at room temperature and the reaction was visualized using Amersham™ ECL™ Western Blotting Detection Reagents (Amersham Pharmaceuticals). Marker proteins were visualized by staining with Brilliant Blue.

### 4.4. Localization of BDNF, TrkB and S100 Protein Using Single and Double Immunofluorescence Staining

To analyze the expression of different proteins in the sensory patches of inner ear, sections were deparaffinized and rehydrated, washed in Phosphate-Buffered Saline (PBS) 0.1 M pH = 7.4 and incubated for 30 min in a PBS solution of fetal bovine serum to avoid non-specific binding, followed by incubation with the primary antibodies. Incubation was carried out overnight at 4 °C in a humid chamber. We used mouse monoclonal antibody pro-BDNF, also designated preproprotein BDNF antibody (Santa Cruz Bio- Biotechnology, CA, USA catalog no. sc-65513) and rabbit polyclonal antibodies against a sequence of amino-terminal mouse BDNF (dilution 1:500; Chemicon International Inc., Temecula, CA, USA; catalog no. AB1534SP), TrkB (dilution 1:500, directed against the residue 794–808 of the intracytoplasmatic domain of human TrkB; Santa Cruz Biotechnology, catalog no. sc-12) and S100 protein directed against bovine S100 protein (Dako, Glostrup, Denmark; code no. Z0311; diluted 1:1000) and it detects both S100A and S100B proteins (manufacturer’s notice) [27,28,48]. After rinsing in PBS, the sections were incubated for 1 h and incubated overnight at 4 °C with Alexa Fluor 488 (Invitrogen, diluted 1:200) and/or with Alexa Fluor 568 (Invitrogen, diluted 1:20) in PBS. Both steps were performed at room temperature in a dark humid chamber. Finally washed, dehydrated and mounted with Fluoromount Aqueous Mounting Medium (Sigma Aldrich, USA). The immunofluorescence was detected using a Zeiss LSMDUO confocal laser scanning microscope with META module (Carl Zeiss Micro Imaging GmbH, Germany) and the images captured were processed using Zen 2011 (LSM 700 Zeiss software). Double fluorescence. Each image was rapidly acquired in order to minimize photodegradation. Digital images were cropped, and the figure montage prepared using Adobe Photoshop 7.0 (Adobe Systems, San Jose, CA, USA). To provide negative controls, representative sections were incubated with specifically preabsorbed antisera as described above. Under these conditions, no positive immunostaining was observed (data not shown).

### 4.5. Scanning Electron Microscopy 

#### Defleshing (Stripping)

Two heads of adult zebrafish utilized for the anatomical study before being fixed, were put in a tank containing tap water for one month in order to accelerate the tissues maceration process [49,50]. During this period the water was changed every two days. Then the samples were fixed in 2.5% glutaraldehyde in Sörensen phosphate buffer 0.1 M. After several rinsing in the same buffer, they were dehydrated in a graded alcohols series, critical-point dried in a Balzers CPD 030, sputter coated with 3 nm gold in a Balzers BAL-TEC SCD 050 and examined under a Zeiss EVO LS 10.2.2 [51,52].

## Supplementary Materials

The following are available online at https://www.mdpi.com/1422-0067/21/16/5787/s1.
