# Supplementary material for: Expression and Localization of BDNF/TrkB System in the Zebrafish Inner Ear
Source: Int J Mol Sci. 2020 Aug 12;21(16):5787. doi: 10.3390/ijms21165787 (PMC7460859; doi:10.3390/ijms21165787)
Supplement: Supplementary file 1 [file ijms-21-05787-s001.pdf]

Supplementary figures:

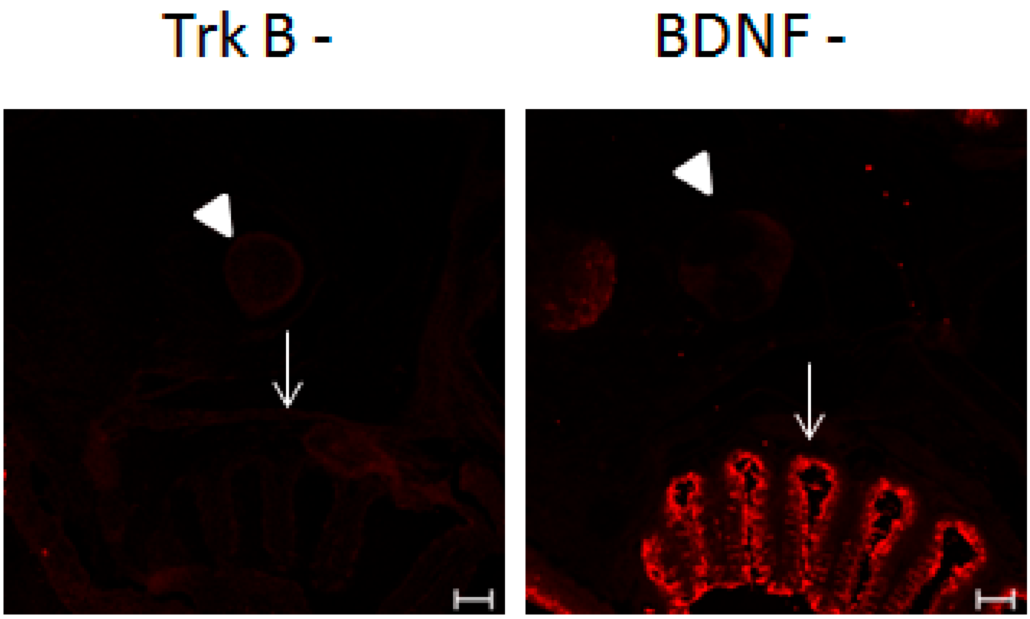

Both negative and positive controls are provided: no immunoreactivity for BDNF and TrkB, using the same slide showed in Figgs. 8,9, 10. Cranial bones are negative for both antibodies (arrowheads) while the olfactory rosetta (arrows) showed immunoreaction only for BDNF. Scale bars= 40 μm

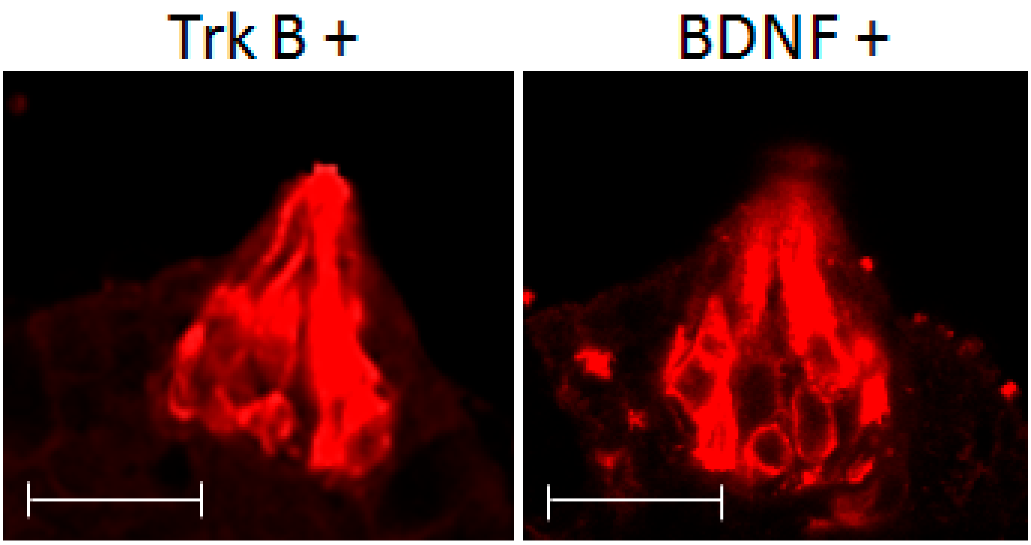

In the same slides used for Fig 8,9,10 immunoreactivity in taste buds for TrkB and BDNF. Scale bars= 20 μm

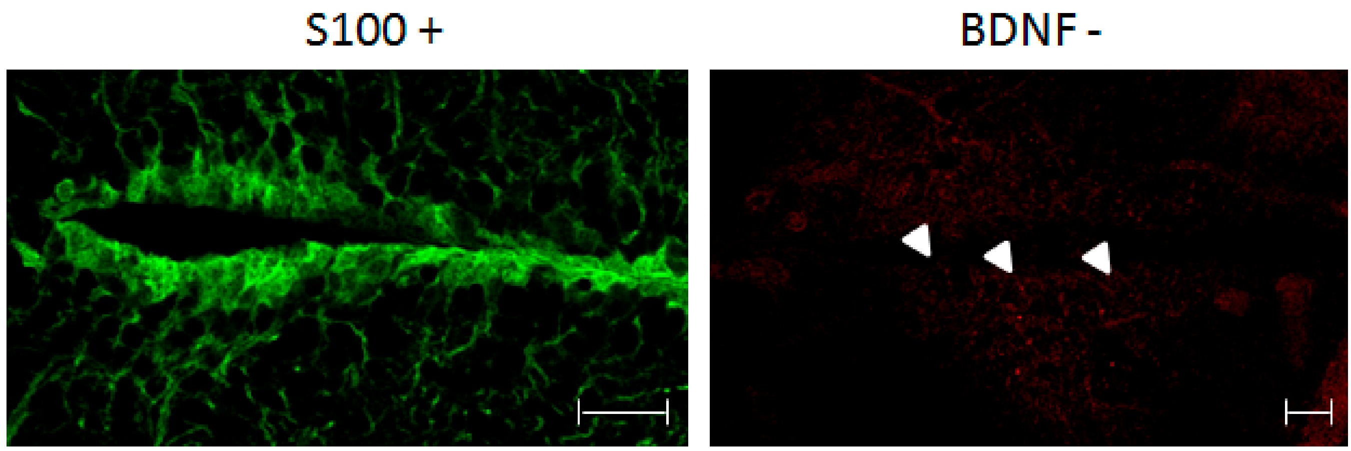

S100+ BDNF- tissue and S100- BDNF- tissue are shown: in the same slides used for Fig 8,9,10, a clear immunoreactivity for S100 in the ependymal cells of diencephalic ventricle while no immunoreactivity is present in the same structure for BDNF (arrowheads). Scale bars= 20 µm

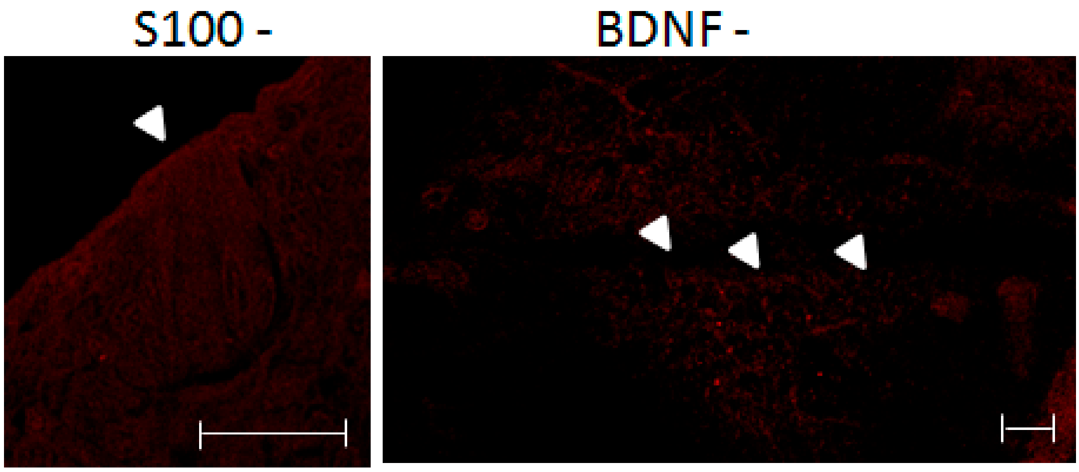

In the same slides used for Fig 8,9,10 no immunoreactivity for S100 in taste buds and for BDNF of diencephalic ventricle ependymal cells. Scale bars= 20 µm
